# A longitudinal network of psychotic-like experiences, depressive and anxiety symptoms, and adverse life events: a cohort study of 3,358 college students

**DOI:** 10.1017/S2045796024000726

**Published:** 2024-11-18

**Authors:** Meng Sun, Heng Sun, Zijuan Ma, Shaoling Zhong, Xinhu Yang, Yue Li, Hongling Zhou, Liang Zhou

**Affiliations:** 1 Department of Social Psychiatry, the Affiliated Brain Hospital, Guangzhou Medical University, Key Laboratory of Neurogenetics and Channelopathies of Guangdong Province and the Ministry of Education of China, Guangzhou Medical University, Guangzhou, China; 2 Department of IT Center, Affiliated Hospital of Jining Medical University, Jining, China; 3 Center for Studies of Psychological Application, School of Psychology, and Guangdong Key Laboratory of Mental Health and Cognitive Science, South China Normal University, Guangzhou, China

**Keywords:** cross-lagged panel network, high-risk population, psychological factors, psychosocial factors, late adolescence

## Abstract

**Aims:**

Psychotic-like experiences (PLEs), especially for persistent PLEs, are highly predictive of subsequent mental health problems. Hence, it is crucial to explore the psychopathological associations underlying the occurrence and persistence of PLEs. This study aimed to explore the above issues through a longitudinal dynamic network approach among PLEs and psychological and psychosocial factors.

**Methods:**

A total of 3,358 college students completed two waves of online survey (from Oct 2021 to Oct 2022). Socio-demographic information was collected at baseline, and PLEs, depressive and anxiety symptoms, and adverse life events were assessed in both waves. Cross-lagged panel network analyses were used to establish networks among individuals with baseline PLEs as well as those without.

**Results:**

At baseline, 455(13.5%) students were screened positive for PLEs. Distinct dynamic network structures were revealed among participants with baseline PLEs and those without. While ‘psychomotor disturbance’ had the strongest connection with PLEs in participants with baseline PLEs, ‘suicide/self-harm’ was most associated with PLEs in those without. Among all three subtypes of PLEs, bizarre experiences and persecutory ideation were the most affected nodes by other constructs in participants with baseline PLEs and those without, respectively. Additionally, wide interconnections within the PLEs construct existed only among participants without baseline PLEs.

**Conclusions:**

The study provides time-variant associations between PLEs and depressive symptoms, anxiety symptoms, and adverse life events using network structures. These findings help to reveal the crucial markers of the occurrence and persistence of PLEs, and shed high light on future intervention aimed to prevent and relieve PLEs.

## Introduction

Psychotic-like experiences (PLEs) refer to experiences similar to positive symptoms of psychosis but which do not cause clinically significant distress, disability or loss of functioning (Kelleher and Cannon, 2011). A volume of research has verified that PLEs in early life are predictive of a series of subsequent mental health problems, including psychotic and non-psychotic disorders (Healy *et al.*, 2019; Kirli *et al.*, 2019), suicide and self-harm (Honings *et al.*, 2016; Yates *et al.*, 2019), reduced global functioning (Calkins *et al.*, 2014), and increased use of mental health service (Bhavsar *et al.*, 2018). Furthermore, these associations are especially stronger for PLEs that persist over time (van Os and Reininghaus, 2016). Hence, PLEs appear to be an important transdiagnostic predictor. And individuals with PLEs, especially for those with persistent PLEs can be regarded as high-risk population of multiple mental disorders.

Given the important roles PLEs play in mental health, it is crucial to explore their risk factors, and thus implementing early prevention and treatment among this population. Previous studies have revealed a battery of risk factors associated with the occurrence of PLEs, including socio-demographic characteristics (e.g. sex [McGrath *et al.*, 2015, 2016b], age, ethnic minority status [Leaune *et al.*, 2019], and urbanicity [DeVylder *et al.*, 2018; Wang *et al.*, 2019]), genetics (Ronald and Pain, 2018), external environment (e.g. childhood trauma and adverse life events) (Bird *et al.*, 2019; Bloomfield *et al.*, 2021) and psychopathology (e.g. depressive and anxiety symptoms) (Bird *et al.*, 2018; Gin *et al.*, 2021). Among those amendable factors, several psychological and psychosocial factors also involve the persistence of PLEs, such as adverse life events 2013, 2011, and depressive and anxiety symptoms (Calkins *et al.*, 2017; Yamasaki *et al.*, 2018). However, due to the limited number of longitudinal studies, current knowledge on persistent PLEs is still sparse, and no predictors of persistent PLEs have been replicated in previous studies (Kalman *et al.*, 2019).

Additionally, PLEs are deemed to be heterogeneous, and different subtypes of PLEs may be involved with different pathogenesis 2012, 2015, 2023. Network analysis provides a novel approach to explore interplay between different subtypes of PLEs and their correlates simultaneously in which psychopathology is conceptualized as a complex dynamic system formed by mutual interactions between symptoms (Robinaugh *et al.*, 2020). So far, a few studies have employed network analysis in unravelling correlates of PLEs on item level (Cheng *et al.*, 2024; Fonseca-Pedrero *et al.*, 2021; Gawęda *et al.*, 2021; Granö *et al.*, 2023; Rejek and Misiak, 2023; Wüsten *et al.*, 2018). However, most of them rely on cross-sectional data, and thus limiting the interpretation of causal interaction as well as the exploration of predictors associated with persistent PLEs.

To better understand the temporal effects of these correlates on PLEs, a longitudinal network approach is warranted. Recently, the cross-lagged panel network (CLPN) analysis, which combines the cross-lagged model and network analysis, has been proposed and used for the exploration of the time-variant associations between individual nodes within the network using longitudinal panel data 2021. We considered it to be an appropriate approach to reveal the psychopathological associations underlying the occurrence and persistence of PLEs. However, to our knowledge, only one research has employed this approach in the association between PLEs and depression and social anxiety in a small sample of psychiatric patients (*N* = 122) (Chavez-Baldini *et al.*, 2022).

To address the above issues, the current study was designed to investigate the dynamic connections between PLEs and psychological and psychosocial factors (including depressive and anxiety symptoms, and adverse life events) using CLPNs in a large sample of college students. According to previous research, the incidence of PLEs peaks in late adolescence or young adulthood (McGrath *et al*. 2016b; Sullivan *et al.*, 2020). College students fall in this age range, and are thought to be a vulnerable population to mental health problems, especially during the COVID-19 pandemic (Huckins *et al.*, 2020; Li *et al.*, 2020), when the data were collected (from Oct 2021 to Oct 2022). In order to explore the dynamic associations associated with the occurrence and persistence of PLEs, network models were established among individuals with baseline PLEs and those without, respectively. We aimed to identify the strongest predictors of PLEs on item levels, as well as the roles of different subtypes of PLEs in the networks. The current study was expected to provide valuable information for understanding the psychopathological mechanisms of the occurrence and persistence of PLEs, and establishing the optimal intervention targeting at PLEs.

## Methods

### Participants and procedure

The study was conducted through convenience sampling among college freshmen from two universities (including 27 colleges) in Guangzhou, China. The participants were invited to complete two waves of online survey, with wave 1 from October to December, 2021, and wave 2 from October to December, 2022. To exclude those with the diagnosis of psychotic disorder at baseline, those scored positive on baseline PLEs (the criteria refer to the Measurements) or had a self-report history of mental disorders at baseline were further invited to receive an in-person structured interview.

The online survey was carried out through delivering the quick response code of questionnaire with the help of the student management office, and the interviews were conducted in the psychological counselling room in campus.

All participants signed the informed consent before each wave of online survey as well as at the beginning of interviews. The investigation was carried out in accordance with the ethical standards of the relevant national and institutional committees on human experimentation and with the Helsinki Declaration of 1975, as revised in 2008 and approved by The Ethics Committees of The Affiliated Brain Hospital of Guangzhou Medical University (2019No.037; 2022No.006).

### Measurements

Socio-demographic information collected by self-report included: age, sex, ethnicity, birth place, chronic physical condition (having at least one of the following: arthritis, angina, asthma, diabetes, visual impairment or hearing problems) (Koyanagi *et al.*, 2018), history of mental disorders and family history of mental disorders.

PLEs were assessed by the 15-item Positive Subscale of the Community Assessment of Psychic Experiences (CAPE-P15) (Capra *et al.*, 2013), covering three subtypes: persecutory ideation (PI), bizarre experiences (BEs) and perceptual abnormalities (PAs) (Nunez *et al.*, 2015). Each item was rated from 1-never, 2-sometimes, 3-often, to 4-nearly always. For the whole scale, the weighted score was adopted through dividing the sum of all item scores by the number of valid items. Score of each subtype can be calculated with the corresponding items. Our previous research has verified the satisfactory psychometric properties of its Chinese version in college students (Sun *et al.*, 2020), and we also identified a weighted score of 1.57 as the optimal cut-off value to detect genuine PLEs using interview-verified PLEs as the golden criteria (Sun *et al.*, 2022). The cut-off value of 1.57 was used to screen participants with baseline PLEs in this study. The scale was used to assess lifetime PLEs and baseline PLEs (in the past month) at wave 1, as well as PLEs in the past year and current PLEs (in the past month) in wave 2. The Cronbach’s ɑ were 0.87, 0.90, 0.88, and 0.89, respectively.

Depressive symptoms were measured by the 9-item Patient Health Questionnaire (PHQ-9) scale. Response to each item ranges from 1-not at all, 2-several days, 3-more than half the days, to 4-nearly every day. The Chinese version of PHQ-9 has been found to be reliable and valid in college students (Zhang *et al.*, 2013). In this study, the scale was used to assess lifetime depressive symptoms (the time window was set at ‘the two weeks in the worst mood in lifetime’) at wave 1, and depressive symptoms in the past year (the time window was set at ‘the two weeks in the worst mood in the past year’) in wave 2. The Cronbach’s ɑ was 0.91 at both waves.

Anxiety symptoms were measured by the 7-item Generalized Anxiety Disorder (GAD-7) scale. It is also a 4-point Likert scale, with each item rated from 1-not at all, 2-several days, 3-more than half the days, to 4-nearly every day. The scale also showed good reliability and validity in Chinese population (Zhang *et al.*, 2021). In this study, the scale was used to assess lifetime anxiety symptoms (the time window was set at ‘the two weeks in the most anxiety mood in lifetime’) at wave 1, and depressive symptoms in the past year (the time window was set at ‘the two weeks in the most anxiety mood in the past year’) in wave 2. The Cronbach’s ɑ was 0.94 at both waves.

Adverse life events were evaluated by the Adolescent Self-rating Life Events Checklist (ASLEC) (Liu *et al.*, 1997), covering six domains: being punished, loss, adjustment, interpersonal stress and academic stress (Xin and Yao, 2015). The scale is consisted of 27 adverse life events during adolescence, and each item was rated from 1-not happened or no effect, 2-mild effect, 3-moderate effect, 4-severe effect, to 5-very severe effect. In this study, the scale was used to assess adverse life events in the past year in both waves. The Cronbach’s ɑ were 0.92 and 0.95, respectively.

The Mini-International Neuropsychiatric Interview for the Diagnostic and Statistical Manual of Mental Disorders, Fifth Edition (DSM-5) (Sheehan *et al.*, 1998; Si *et al.*, 2009) was used to screen psychotic disorders among participants who scored above 1.57 for baseline PLEs and self-reported a history of mental disorders.

### Statistical analysis

All analyses were performed using R (V.4.2.3). A two-sided *p*-value < .05 was considered statistically significant. To ensure the quality of online survey, we excluded those with the response time less than 5 minutes during each wave of the survey. Additionally, those with a history of psychotic disorders were also excluded, as PLEs refer to those symptoms in the absence of psychotic disorders.

We first compared socio-demographic characteristics and baseline clinical variables (i.e. PLEs, depressive and anxiety symptoms, and adverse life events) between participants who follow-up and those lost to follow-up. Participants with valid data in both waves of survey were retained for the following analysis.

Given that different dynamic network systems may exist in participants with and without baseline PLEs, we divided the whole sample into two groups according to the cut-off value of 1.57, and then explored the interplay of PLEs, depressive and anxiety symptoms, and adverse life events using CLPN analysis in the two groups, respectively. Socio-demographic characteristics and two waves of clinical data were also compared between the two groups of participants.

The CLPNs were estimated with autoregressive and cross-lagged coefficients by performing a series of regularized regressions using the R-package *glmnet* (Funkhouser *et al.*, 2021). The R-package *qgraph* (Epskamp *et al.*, 2018) was used to visualize the CLPNs. Four constructs including 24 symptoms (PLEs: three factors of CAPE-P15; depressive symptoms: nine items of PHQ-9; anxiety symptoms: seven items of GAD-7; adverse life events: five factors of ASLEC) constituted the networks. In a CLPN graph, nodes represent symptoms. The colour and thickness of the edge reflect the cross-lagged coefficients (from wave 1 to wave 2), with red indicating negative values, blue representing positive values, and thicker edges indicating higher absolute weight values.

Two centrality indices were used to evaluate centrality, among which, out expected influence (out-EI) is calculated by the sum of all absolute values of all edges coming from the node, and in expected influence (in-EI) is the sum of all absolute values of all edges pointing to the node. Out-EI and in-EI reflect the degree of the effect of a node towards and from other nodes, respectively. Additionally, as we focused on the connections between PLEs construct and other constructs in the current study, the bridge-EIs were also calculated with the R-package *networktools*, to reflect the how much influence a node has on and from other constructs (bridge out-EI and bridge in-EI) (Jones *et al.*, 2021). All autoregressive effects were removed when calculating centrality. To compare the two networks, we conducted a matrix correlation analysis between edge weight matrices of the two groups to evaluate the similarities between the network structures, and calculated the proportion of edges in the network of the baseline PLEs group that replicated in the network of those without baseline PLEs. Pearson correlation analyses were also conducted between the two networks in centrality indices, including out-EI, in-EI, bridge out-EI and bridge in-EI. To focus on connections between nodes, autoregressive effects were removed when comparing centrality indices of the two networks (Funkhouser *et al.*, 2021).

The accuracy and stability of CLPNs were evaluated following the steps in the R-package *boonet* (Epskamp *et al.*, 2018). First, a nonparametric bootstrapping procedure with 1,000 iterations was used to assess the 95% confidence intervals (CIs) of each edge weight, with larger indicating poorer precision in the estimation of edges. And then the correlation stability (CS) coefficients were calculated by case-drop bootstrapping to determine the stability of the estimations of the centrality indices. The CS ranges from 0 to 1, with no less than 0.25 representing acceptable stability and more than 0.5 representing strong stability. To better avoid the influence of other factors, all socio-demographic characteristics were included as covariates in the CLPN models.

## Results

### Description of the sample

**Figure 1.**
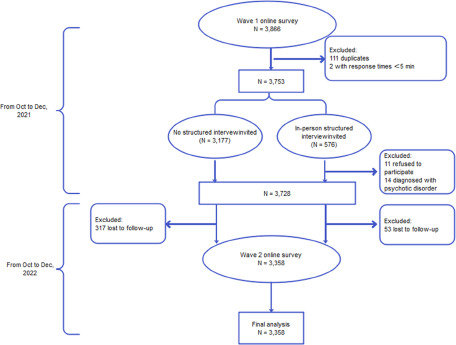
Flow of procedure.

A total of 3,866 questionnaires were collected at wave 1, and 576 participants were invited to in-person clinical interview. After excluding 111 duplicates (the first versions were adopted), 2 with response time less than 5 minutes, 14 with a history of psychotic disorder, and 11 who refused to participate in the in-person interviews, 3,728 participants provided valid data at baseline. At wave 2,370 participants lost to follow-up, and a total of 3,358 participants were included in the final analysis. Details of the procedure see Figure 1.

No significant differences were found in socio-demographic characteristics or clinical assessments in comparisons of participants retained and those lost to follow-up (all *p* > .05). According to the cut-off values of 1.57 for baseline PLEs, 455 (13.5%) participants were identified as positive. Table 1 displays the baseline information of participants with baseline PLEs and those without. Compared to participants without baseline PLEs, higher proportion of those with baseline PLEs were born in rural (*p* = .031), and had chronic physical conditions (*p* *<* .001) and history of mental disorders (*p <* .001). Additionally, they scored significantly higher on all PLEs, depressive and anxiety symptoms, and adverse life events than their counterparts in both waves of survey (all *p* *<* .001). Additionally, 22.9% (*N* = 104) of the participants with baseline PLEs continued to experience these symptoms at wave 2, while 4.7% (*N* = 137) of those without baseline PLEs suffered from PLEs.

**Table 1.** Socio-demographic and clinical variables

	Participants with baseline PLEs (*N* = 455)	Participants without baseline PLEs (*N* = 2,903)	*t*/χ^2^	*p*-value
***Socio-demographic variables [M(SD)/N(%)]***				
Age	18.8 (0.62)	18.8 (0.69)	−0.766	.444
Sex (female)	252 (55.4)	1586 (54.6)	0.090	.800
Ethnicity (Han^a^)	445 (97.8)	2,804 (96.6)	1.841	.201
Birth place				
Urban	125 (27.5)	957 (33.0)	6.935	.031
Town	121 (26.6)	781 (26.9)		
Rural	209 (45.9)	1,165 (40.1)		
Chronic physical condition^b^ (Yes)	59 (13.0)	224 (7.7)	14.053	<.001
History of mental disorders (Yes)	25 (5.5)	43 (1.5)	31.933	<.001
Family history of mental disorders (Yes)	28 (6.2)	79 (2.7)	15.024	<.001
***Clinical variables [N(%)]***				
PLEs at wave1	1.97 (0.26)	1.33 (0.24)	49.380	<.001
PLEs at wave2	1.41 (0.39)	1.19 (0.23)	11.732	<.001
PHQ-9 at wave1	11.05 (5.66)	6.06 (4.59)	16.722	<.001
PHQ-9 at wave2	7.94 (5.69)	4.79 (4.17)	10.958	<.001
GAD-7 at wave1	9.18 (5.20)	4.88 (4.39)	17.886	<.001
GAD-7 at wave2	6.19 (4.88)	3.59 (3.93)	11.323	<.001
ASLEC at wave1	47.01 (14.02)	36.16 (9.63)	15.922	<.001
ASLEC at wave2	42.58 (14.81)	34.85 (10.82)	10.696	<.001

Note: PLEs, psychotic-like experiences; PHQ-9, the 9-item Patient Health Questionnaire; GAD-7, the 7-item Generalized Anxiety Disorder scale; ASLEC, the Adolescent Self-rating Life Event Checklist.

a Han is the major ethnic group in China.

b Chronic physical conditions referred to having at least one of arthritis, angina, asthma, diabetes, visual impairment or hearing problems.

### Longitudinal networks of PLEs, depressive symptoms, anxiety symptoms and adverse life events

**Figure 2.**
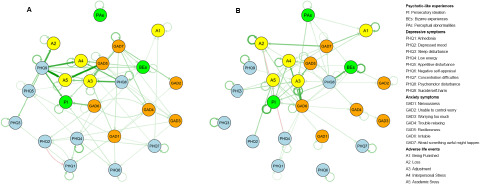
Longitudinal networks of psychotic-like experiences, depressive symptoms, anxiety symptoms, adverse life events and perceived stress.

The temporal networks of the two groups are shown in Fig. 2. The edge weight matrix used to plot the CLPNs can be found in Supplementary materials_Table S1–2. All three subtypes of PLEs showed significant edges from wave 1 to wave 2, with PI having greatest autoregression coefficients in both groups (Group with baseline PLEs: *Edge weight* = .138; Group without baseline PLEs: *Edge weight* = .243). Within the PLEs construct, no associations were found among the three subtypes in the baseline PLEs group, while there were a wide range of connections in those without baseline PLEs. As for connections between PLEs and other constructs, the strongest edges in the group with baseline PLEs were from PHQ-9 (suicide/self-harm) to PI (*Edge weight* = .205), from GAD-6 (irritable) to PI (Edge weight = .174), and from GAD-5 (restlessness) to BEs (*Edge weight* = .106), while the edges from PHQ-8 (psychomotor disturbance) to BEs (*Edge weight* = .217), from PHQ-8 (psychomotor disturbance) to PI (*Edge weight* = .103), and from GAD-6 (irritable) to PI (*Edge weight* = .088) showed greatest cross-lagged coefficients in those without baseline PLEs. Regarding to the connections between PLEs and adverse life events, the strongest edge was from A3 (adjustment) to PI (*Edge weight* = .034) in the group without baseline PLEs, followed by A3 to BEs (*Edge weight* = .022), with no edge being found from adverse life events to PLEs in the baseline PLEs group. However, there were several edges from A4 (interpersonal stress) to other constructs, such as A4 to PHQ-7 (concentration difficulties) (*Edge weight* = .016) and A4 to GAD-3 (worrying too much) (*Edge weight* = .018).

A. Network among participants with baseline psychotic-like experiences (*N* = 455); B. Network among participants without baseline psychotic-like experiences (*N* = 2,903). Note: Arrows represent longitudinal association between different nodes. Elements for different constructs are in different colours. Nodes with stronger connection are automatically clustered together by the Fruchterman–Reingold algorithm. Edges with weights lower than 0.05 were excluded to ease visual interpretation. Both networks have controlled for covariates, including age, sex, ethnicity, birth place, chronic physical condition, history of mental disorders and family history of mental disorders

**Figure 3.**
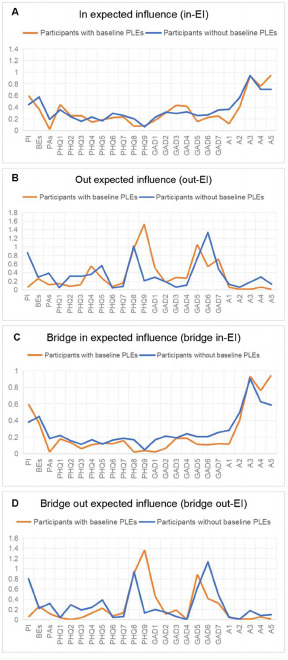
Centrality and bridge centrality indices for nodes in networks of participants with baseline PLEs and those without.

Figure 3 displays centrality estimates of all 24 nodes. A3 (adjustment) had highest in-EI (0.934 and 0.941) in both groups, while PAs showed the lowest in-EI (0.020) in the group with baseline PLEs, and PHQ-9 (suicide/self-harm) had the lowest in-EI (0.063) in participants without baseline PLEs. In the baseline PLEs group, PHQ-9 (suicide/self-harm) had the highest out-EI (1.529), followed by GAD-5 (restlessness, 1.056), while A3 (adjustment) had the lowest out-EI (0.008). In those without baseline PLEs, GAD-6 (irritable, 1.335), PHQ-8 (psychomotor disturbance, 1.015) and PI (0.861) had relatively high out-EI compared to other nodes, while PHQ-6 (negative self-appraisal) had lowest out-EI (0.044). Bridge centrality indices were also computed to reflect centrality between constructs. For PLEs, PI (0.598 and 0.381) and BEs (0.368 and 0.448) had higher bridge in-EI than PAs (0.020 and 0.184) in both groups. Furthermore, PAs showed the lowest bridge in-EI (0.020) of all nodes in the baseline PLEs group. In regard to bridge out-EI, BEs (0.261) and PAs (0.118) had higher estimations than PI (0.054) in the baseline PLEs group, while PI (0.803) showed the highest bridge out-EI of all three PLEs subtypes in those without baseline PLEs.

A. In expected influence; B. Out expected influence; C. Bridge in expected influence; D. Bridge out expected influenceNote: Higher values indicate more centrality. Autoregressive effects are removed when calculating the indices. PLEs, psychotic-like experiences; PI, persecutory ideation; BEs, bizarre experiences; PAs, perceptual abnormalities; PHQ-1–9, 9 items of the Patient Health Questionnaire; GAD-1-7, seven items of the Generalized Anxiety Disorder scale; ASLEC-1–5, five factors of the Adolescent Self-rating Life Events Checklist

The network structures of the two groups exhibited quite a low correlation (*r* = .132, *p* = .032). Only 29.3% (*n* = 89) of the edges in the network of the baseline PLEs group were replicated in the network of those without baseline PLEs. Regarding centrality indices, both in-EI and bridge in-EI are highly correlated (*r* = .878 and .921, both *p* *<* .001), while no significant relationship was found in out-EI or bridge out-EI (*p* = .101 and .056).

### Accuracy and stability of the CLPNs

Bootstrapped 95% CIs of the edge weights of the two networks were relatively narrow, indicating good accuracy for the CLPNs (see Supplementary materials_Figure S1). Edge weight differences and centrality differences are displayed in Supplementary materials_Figure S2 and 3. The estimations of out-EI, in-EI and bridge-EI showed acceptable stability in both groups in case-drop bootstrapping (see Supplementary materials_Figure S4). The CS coefficients for out-EI, in-EI and bridge-EI were 0.26, 0.51 and 0.26, respectively in the group with baseline PLEs, and 0.49, 0.75 and 0.49, respectively in those without baseline PLEs.

## Discussion

To our knowledge, this is the first study to explore the longitudinal relationships between PLEs and psychological and psychosocial factors using network modelling. In this cohort of college students, ‘psychomotor disturbance’ was identified as the strongest predictor of the severity of PLEs for individuals without baseline PLEs, and ‘suicide/self-harm’ was most closely associated with the persistence of PLEs. Among adverse life events, ‘adjustment’ exerted an influence on the subsequent severity of PLEs among those without PLEs at baseline, while ‘interpersonal stress’ may affect the persistence of PLEs indirectly through depressive and anxiety symptoms. Among all three subtypes of PLEs, BEs received the highest degree of effects from other constructs when PLEs score was below the cut-off value, while it exerted strongest influence when PLEs score was above the cut-off value. The opposite is the case for PI. Additionally, a high degree of interconnections within the PLEs construct existed only before the threshold score of PLEs was reached.

Comparisons between the two networks (the low-level correlation of the two network structures and no significant correlation of out-EI or bridge out-EI) reveal that different patterns of interaction between PLEs and other psychological and psychosocial symptoms may exist before and after the occurrence of PLEs. Among those without baseline PLEs, the strongest edge linked to PLEs was from ‘psychomotor disturbance’, which is the core feature of melancholic depression (APA, 2013), and usually represents the more severe end of the depression continuum (Parker *et al.*, 2017). Furthermore, previous studies have revealed the higher level of severity of psychomotor disturbance in psychotic depression than that in non-psychotic depression, both during acute episodes and in remission (Bingham *et al.*, 2021; Fleming *et al.*, 2004). Potential pathophysiological mechanisms of psychomotor disturbances are related to abnormal emotional-motor processing in prefrontal cortical networks across schizophrenic and affective psychosis (Northoff *et al.*, 2004). In this network, the temporal relationship of ‘psychomotor disturbance’ and PLEs may suggest that this symptom may be interpreted as a trait marker predisposing for subsequent psychosis. Exploration of the underlying mechanism is also needed in this subclinical stage. The node most strongly connected to PLEs was ‘suicide/self-harm’ in the baseline PLEs group. Compared to most research which usually treat suicide and self-injurious behaviour as the outcome of PLEs (Honings *et al.*, 2016; Yates *et al.*, 2019), only a small number of studies have verified the reverse prediction (Murphy *et al.*, 2022; Zhou *et al.*, 2024). According to the suicidal drive hypothesis (Murphy et al., 2018b), PLEs might reflect an individual’s thoughts in the context of internal threat exposure (i.e. suicide/self-harm). By attributing internal threat to sources external to themselves, individuals experiencing suicide/self-harm may protect themselves from their own thoughts or behaviours that have become an imminent mortality risk. Our results reconfirmed the hypothesis in a relatively large sample. We further found that this reverse prediction only existed in the group with baseline PLEs, which reminds the practitioners to be alert to psychosis risk among this population and to consider more intense intervention that can help them from developing more complex mental health problems. Additionally, ‘irritable’ seemed to be a shared predictor for both the occurrence and persistence of PLEs, which is in line with previous research using regression analysis (Morales-Muñoz *et al.*, 2022), that persistent high levels of anxiety in early life could be a risk factor for psychosis via activation of stress hormones and nonresolving inflammation. Our results contribute to future preventive interventions in psychosis, with specific anxiety symptom for target.

Although previous research has verified the predictive effects of depressive and anxiety symptoms on PLEs (Hartley *et al.*, 2013), comprehensive interactions between single symptoms are inaccessible from traditional regression analysis. Meanwhile, due to the bidirectional associations between PLEs and emotional symptoms (Healy *et al.*, 2020; McGrath *et al.*, 2016a), previous network using cross-sectional data failed to determine their sequence. By utilizing temporal network modelling, the current study revealed that different emotional symptoms respectively played leading roles in the occurrence and persistence of PLEs, suggesting different targets should be focused on according to the phase of the development of PLEs.

In respect to adverse life events, ‘adjustment’ appeared to be the solely significant predictor for PLEs in the group without baseline PLEs. In the current study, all participants were college freshmen, who had to face a series of adaptation problems, such as academic, interpersonal and campus life adjustment (Cao, 2022; English *et al.*, 2017). A longitudinal study has verified the positive correlation between adjustment and mental health, including emotional symptoms and subjective well-being (Zhang *et al.*, 2023). Our results further revealed that college adjustment could also increase the severity of PLEs for those without PLEs at baseline. Meanwhile, there were no direct effects from adverse life events to PLEs in the baseline PLEs group. Interpersonal stress exhibited negative effects on depressive and anxiety symptoms, and may exert an influence on PLEs through these emotional symptoms. These findings emphasize the important role of adverse life events in the occurrence and development of both emotional symptoms and PLEs, and suggest to bring more attention to the adjustment problems and interpersonal stress in college freshmen. Several intervening measures acting on these targets are expected to improve overall mental health among this population, including group activities that help to adapt to campus life, courses on improving social skills, and mindful training aimed to reduce stress.

The most central symptoms bridging PLEs and other constructs also differed among the two network structures. In the network of individuals without baseline PLEs, BEs had the highest bridge in-EI (0.448), and PI had highest bridge out-EI (0.803). However, in the network among the baseline PLEs group, PI showed the highest bridge in-EI (0.598), and BEs showed the highest bridge out-EI (0.261). Both BEs (Fonseca-Pedrero *et al.*, 2021; Yang *et al.*, 2023) and PI (Murphy *et al.*, 2018a) have been found to be the most central node in cross-sectional network analysis. However, the current study first found the different roles of PI and BEs played in the occurrence and persistence of PLEs. The extensive influence of PI in individuals without PLEs, as well as and its most susceptible role in the PLEs group, suggest PI be a non-specific transdiagnostic indicator. Meanwhile, the role transformation of BEs from the affected node to the influencer implies that BEs play a pivotal role in the phase of development of PLEs. In both network structures, PAs received the lowest influence from other constructs and other PLEs subtypes, indicating that PAs were less likely to be amended through these psychological and psychosocial factors as well as other PLEs subtypes. The distinct roles of PI, BEs and PAs in the network structures further confirm PLEs to be a heterogenic phenomenon (Remberk, 2017), and indicate that different subtypes are based on distinct psychopathological underpinnings.

Additionally, there were wide links among the three subtypes within the PLEs construct among individuals without baseline PLEs, which provide evidence for the network-type dynamic interactions within the PLEs construct during the development of PLEs (van Os and Reininghaus, 2016). Of all three subtypes, BEs exhibited the widest links with other PLEs subtypes, indicating that BEs affect other psychological and psychosocial factors both directly and indirectly (through other PLEs subtypes) at the initial phase of PLEs. The dynamic associations within the PLEs construct also differ when PLEs score was below and above the threshold value. Unlike the wide interconnections in participants without baseline PLEs, no connections were found among PI, BEs and PAs in the baseline PLEs group. The results suggest that heterogeneity among different subtypes may further increase since the occurrence of PLEs and lead to the diversity of clinical outcomes. Future research should consider to connect these different subtypes to subsequent clinical outcomes.

## Limitations

Several limitations need to be clarified when interpreting the results. First, as an epidemiological study, no genetic data were collected in this study. Although we included family history of mental disorders as covariate in the analysis, it cannot be equal to genetic aetiology as it can also be related to other reasons (e.g. shared environment). Second, several correlates which may also exert influence on PLEs were not included in the current analysis, such as cognitive deficits (Gawęda *et al.*, 2021) and frequent cannabis use (Bourque *et al.*, 2017). Considering the sample size, we only chose correlates with robust evidence in the current study. More comprehensive factors should be considered in future research. Third, although the CLPNs assessed the temporal relationship among PLEs, depressive and anxiety symptoms, and adverse life, it cannot be equal to causality. Further research is needed to reveal the underlying biological and psychological mechanism in future. Additionally, it should be noted that most of the centrality indices were far below 0.5 in the group with baseline PLEs, which failed to achieve the criteria for strong stability. The results may be attributed to the small sample size in this group (*N* = 455), and need to be validated in larger sample. Furthermore, we found little effects from adverse life event in the current study, which may result from the population we chose. As all participants were well-educated college students, most of them were from families with good socio-economic status and less affected by adverse life events. Hence, these findings need to be interpreted with caution when generalized to other population. Finally, the convenience sampling method may also limit the representative of the sample. Further validation of these findings is needed in a representative sample of college students.

## Conclusions

Overall, the study provides time-variant associations between PLEs and depressive symptoms, anxiety symptoms, and adverse life events using longitudinal panel data. Different patterns of interaction between PLEs and other psychological and psychosocial symptoms were identified during the different phases of the development of PLEs. The findings help to specify the crucial markers of occurrence and persistence of PLEs, as well as shed high light on future intervene targets and measures to prevent the onset of PLEs as well as to relieve symptoms in individuals with PLEs.

## Clinical implications

The study highlights the distinct dynamic network structures during the development of PLEs. At initial phase, early intervention should be focused on those with high level of psychomotor disturbance in order to prevent the occurrence of PLEs. Several treatments, such as mindfulness and physical exercise, have been found to be effective in reducing relevant symptoms, and should be taken to consideration in promoting campus mental health. For students screened positive for PLEs, more attention should be paid to those with comorbid suicide or self-harm, as they are not only individuals with risk behaviour, but high-risk population for subsequent psychosis. Besides, irritable should also be treated as an important target for early intervention both for those with and without PLEs. Finally, intervening measures should also be taken to help college students better adapt to campus life as well as reduce interpersonal stress.

## Supporting information

Sun et al. supplementary material 1 Sun et al. supplementary material

Sun et al. supplementary material 2 Sun et al. supplementary material

Sun et al. supplementary material 3 Sun et al. supplementary material

Sun et al. supplementary material 4 Sun et al. supplementary material

Sun et al. supplementary material 5 Sun et al. supplementary material

Sun et al. supplementary material 6 Sun et al. supplementary material

## Data Availability

The data that support the findings of this study are available on request from the corresponding author, [L. Zhou].

## Supplementary material

The supplementary material for this article can be found at https://doi.org/10.1017/S2045796024000726.
