# Supplementary figures and images for: A longitudinal network of psychotic-like experiences, depressive and anxiety symptoms, and adverse life events: a cohort study of 3,358 college students
Source: Epidemiol Psychiatr Sci. 2024 Nov 18;33:e64. doi: 10.1017/S2045796024000726 (PMC11669803; doi:10.1017/S2045796024000726)

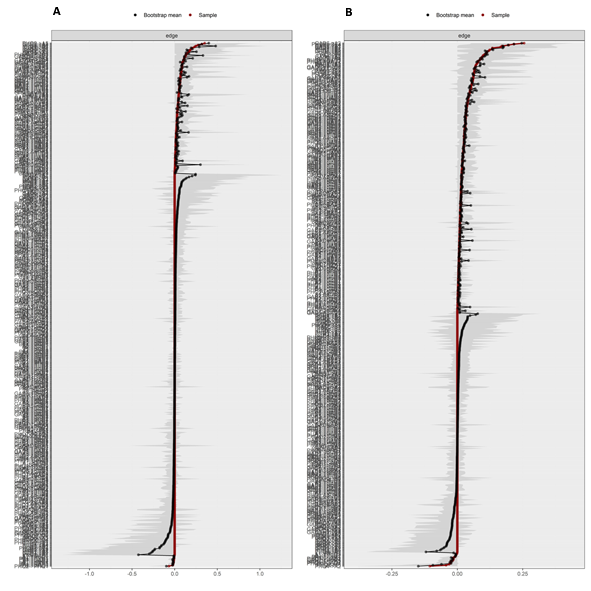

Supplement: Sun et al. supplementary material 1 — Sun et al. supplementary material [file S2045796024000726sup001.tiff]

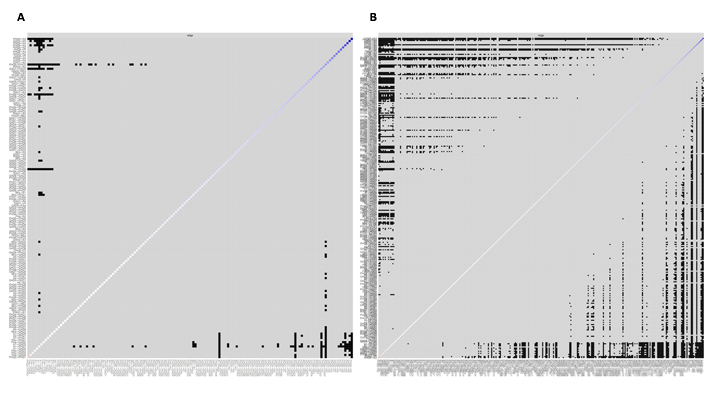

Supplement: Sun et al. supplementary material 2 — Sun et al. supplementary material [file S2045796024000726sup002.tiff]

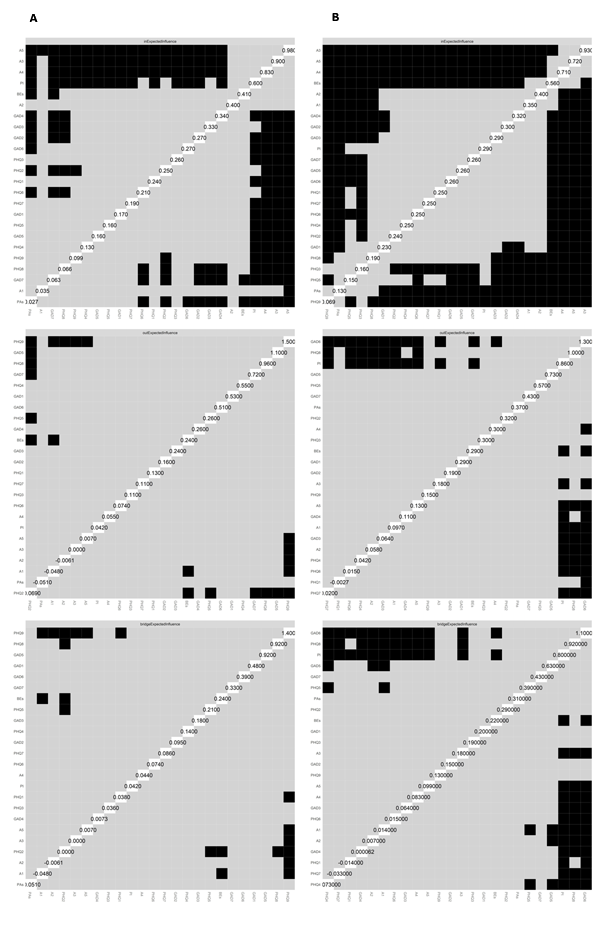

Supplement: Sun et al. supplementary material 3 — Sun et al. supplementary material [file S2045796024000726sup003.tiff]

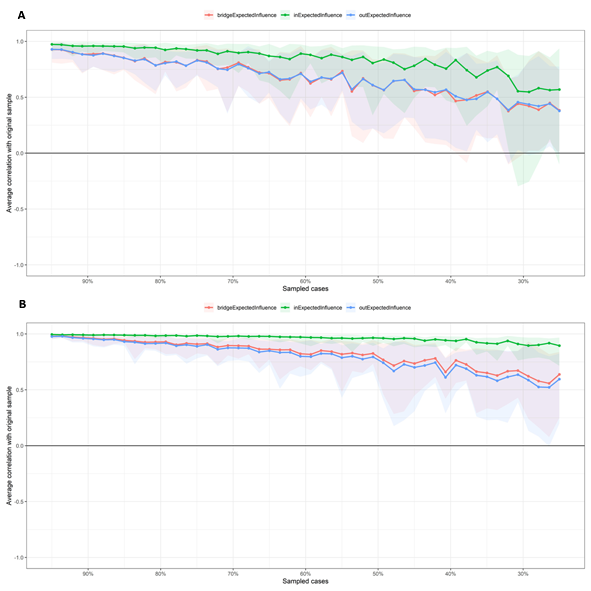

Supplement: Sun et al. supplementary material 4 — Sun et al. supplementary material [file S2045796024000726sup004.tiff]
