# Supplementary material for: A longitudinal network of psychotic-like experiences, depressive and anxiety symptoms, and adverse life events: a cohort study of 3,358 college students
Source: Epidemiol Psychiatr Sci. 2024 Nov 18;33:e64. doi: 10.1017/S2045796024000726 (PMC11669803; doi:10.1017/S2045796024000726)
Supplement: Sun et al. supplementary material 5 — Sun et al. supplementary material [file S2045796024000726sup005.doc]

Figure S1. Bootstrapped confidence intervals of the edge weights of the networks in two groups

1. Participants with baseline PLEs; B. Participants without baseline PLEs

Note: The wider the bootstrapped CI is for one edge, the more careful the inferences should be.

Figure S2. Edge weight differences for the networks of the two groups

1. Participants with baseline PLEs; B. Participants without baseline PLEs

Note: Black box represents an edge that differs significantly (α = 0.05) from another one.

Figure S3. Node centrality differences for the network of the two groups

1. Participants with baseline PLEs; B. Participants without baseline PLEs

Note: Black box represents an edge that differs significantly (α = 0.05) from another one.

Figure S4. Centrality stability in the networks of the two groups

1. Participants with baseline PLEs; B. Participants without baseline PLEs

Note: Average correlations between different centrality indices sampled with persons dropped and the original sample. Lines indicate the means and areas indicate the range from the 2.5th quantile to the 97.5th quantile.
