# Supplementary material for: A longitudinal network of psychotic-like experiences, depressive and anxiety symptoms, and adverse life events: a cohort study of 3,358 college students
Source: Epidemiol Psychiatr Sci. 2024 Nov 18;33:e64. doi: 10.1017/S2045796024000726 (PMC11669803; doi:10.1017/S2045796024000726)
Supplement: Sun et al. supplementary material 6 — Sun et al. supplementary material [file S2045796024000726sup006.doc]

Table S1. Edge weights matrix for the network of participants with baseline PLEs

|  | PI | BEs | PAs | PHQ1 | PHQ2 | PHQ3 | PHQ4 | PHQ5 | PHQ6 | PHQ7 | PHQ8 | PHQ9 | GAD1 | GAD2 | GAD3 | GAD4 | GAD5 | GAD6 | GAD7 | A1 | A2 | A3 | A4 | A5 |
| --- | --- | --- | --- | --- | --- | --- | --- | --- | --- | --- | --- | --- | --- | --- | --- | --- | --- | --- | --- | --- | --- | --- | --- | --- |
| PI | 0.138 | 0 | 0 | 0 | 0 | 0 | 0 | 0.002 | 0 | 0 | 0 | 0.038 | 0 | 0 | 0 | 0 | 0 | 0 | 0.014 | 0 | 0 | 0 | 0 | 0 |
| BEs | 0 | 0.091 | 0 | 0.030 | 0 | 0.005 | 0 | 0 | 0 | 0 | 0.006 | 0 | 0 | 0 | 0.004 | 0.019 | 0.013 | 0 | 0.022 | 0.053 | 0 | 0.056 | 0.014 | 0.040 |
| PAs | 0 | 0 | 0.102 | -0.009 | 0 | 0 | 0 | 0 | 0 | 0 | 0 | 0 | 0 | -0.013 | -0.023 | -0.024 | 0 | 0 | -0.023 | 0 | 0.026 | 0 | 0 | 0 |
| PHQ1 | 0 | 0 | 0 | 0.121 | 0.064 | 0 | 0.034 | 0 | 0.005 | 0 | 0 | 0 | 0 | 0 | 0.036 | 0.002 | 0 | 0 | 0 | 0 | 0 | 0 | 0 | 0 |
| PHQ2 | 0 | 0 | 0 | -0.082 | 0 | 0 | 0 | 0 | 0 | 0 | 0 | 0 | 0 | 0 | 0 | 0 | 0 | 0 | 0 | 0 | 0 | 0 | 0 | 0 |
| PHQ3 | 0 | 0 | 0 | 0.066 | 0 | 0.124 | 0 | 0 | 0 | 0 | 0.011 | 0 | 0 | 0 | 0 | 0 | 0 | 0 | 0 | 0 | 0 | 0.038 | 0 | 0 |
| PHQ4 | 0 | 0.062 | 0 | 0.091 | 0.056 | 0.029 | 0.178 | 0.058 | 0.076 | 0.073 | 0.043 | 0 | 0.004 | 0.013 | 0 | 0.026 | 0 | 0.025 | 0 | 0 | 0 | 0 | 0 | 0 |
| PHQ5 | 0.017 | 0.011 | 0 | 0 | 0 | 0.026 | 0 | 0.058 | 0 | 0 | 0 | 0.019 | 0 | 0.005 | 0 | 0 | 0 | 0 | 0.018 | 0 | 0.128 | 0.050 | 0 | 0 |
| PHQ6 | 0.074 | 0 | 0 | 0 | 0 | 0 | 0 | 0 | 0.071 | 0 | 0 | 0 | 0 | 0 | 0 | 0 | 0 | 0 | 0 | 0 | 0 | 0 | 0 | 0 |
| PHQ7 | 0 | 0 | 0 | 0.025 | 0 | 0 | 0 | 0 | 0 | 0.132 | 0 | 0 | 0 | 0.014 | 0 | 0 | 0 | 0 | 0 | 0.063 | 0 | 0 | 0.055 | 0 |
| PHQ8 | 0 | 0 | 0 | 0 | 0 | 0.007 | 0 | 0 | 0 | 0 | 0.150 | 0.025 | 0.003 | 0 | 0.063 | 0.088 | 0.080 | 0.056 | 0.036 | 0 | 0 | 0.240 | 0.187 | 0.159 |
| PHQ9 | 0.205 | 0.100 | 0 | 0.003 | 0 | 0.135 | 0 | 0 | 0.021 | 0 | 0 | 0.170 | 0.014 | 0.021 | 0.009 | 0 | 0.020 | 0.027 | 0 | 0 | 0.247 | 0.112 | 0.268 | 0.347 |
| GAD1 | 0 | 0.001 | 0 | 0.079 | 0 | 0 | 0 | 0.064 | 0.066 | 0.052 | 0 | 0 | 0.040 | 0.051 | 0 | 0 | 0 | 0 | 0 | 0 | 0 | 0.089 | 0.064 | 0.045 |
| GAD2 | 0 | 0 | 0 | 0 | 0 | 0 | 0.014 | 0.035 | 0 | 0.038 | 0 | 0 | 0 | 0 | 0 | 0 | 0.024 | 0.044 | 0 | 0 | 0 | 0.020 | 0 | 0 |
| GAD3 | 0 | 0 | 0 | 0 | 0.063 | 0.027 | 0.024 | 0.019 | 0.056 | 0 | 0 | 0 | 0.010 | 0 | 0.0004 | 0.042 | 0 | 0 | -0.046 | 0 | 0 | 0 | 0 | 0 |
| GAD4 | 0 | 0 | 0 | 0 | 0 | 0.007 | 0 | 0 | 0 | 0 | 0 | 0 | 0.064 | 0.033 | 0.087 | 0 | 0.004 | 0.072 | 0 | 0 | 0 | 0 | 0 | 0 |
| GAD5 | 0.101 | 0.106 | 0.020 | 0 | 0 | 0.009 | 0 | 0 | 0 | 0 | 0.008 | 0 | 0 | 0.025 | 0.058 | 0 | 0.096 | 0.001 | 0.084 | 0 | 0 | 0.188 | 0.145 | 0.313 |
| GAD6 | 0.174 | 0 | 0 | 0.045 | 0.070 | 0.015 | 0.061 | 0.006 | 0 | 0.037 | 0.006 | 0 | 0 | 0 | 0.042 | 0.083 | 0 | 0.061 | 0 | 0 | 0 | 0 | 0 | 0 |
| GAD7 | 0.028 | 0.088 | 0 | 0 | 0 | 0 | 0 | 0 | 0 | 0 | 0 | 0 | 0.079 | 0.128 | 0.062 | 0.107 | 0.011 | 0.001 | 0.162 | 0 | 0 | 0.140 | 0.027 | 0.042 |
| A1 | 0 | 0 | 0 | -0.008 | 0 | 0 | 0 | 0 | 0 | -0.016 | 0 | 0 | 0 | 0 | -0.019 | -0.015 | 0 | 0 | 0 | 0.082 | 0 | 0 | 0 | 0 |
| A2 | 0 | 0 | 0 | -0.007 | 0 | 0 | 0 | 0 | 0 | 0 | 0 | 0 | 0 | 0 | 0.006 | 0 | 0 | 0 | 0 | 0 | 0.108 | 0 | 0 | 0 |
| A3 | 0 | 0 | 0 | 0 | 0 | 0 | 0 | 0 | 0 | 0 | 0 | 0 | 0 | 0 | 0 | 0 | 0 | 0 | 0.008 | 0 | 0 | 0.116 | 0 | 0 |
| A4 | 0 | 0 | 0 | 0.001 | 0 | 0 | 0.010 | 0 | 0 | 0.016 | 0 | 0 | 0 | 0 | 0.018 | 0.012 | 0 | 0 | 0 | 0 | 0 | 0 | 0.135 | 0.008 |
| A5 | 0 | 0 | 0 | 0 | 0 | 0 | 0 | 0 | 0 | 0 | 0 | 0 | 0 | 0 | 0.009 | 0 | 0 | 0 | 0 | 0 | 0 | 0 | 0 | 0.109 |

Note: Variables at wave 2 are in rows and variables at wave 1 are in columns.

PLEs, psychotic-like experiences; PI, persecutory ideation; BEs, bizarre experiences; PAs, perceptual abnormalities; PHQ1-9, 9 items of the Patient Health Questionnaire; GAD1-7, seven items of the Generalized Anxiety Disorder scale; A1-5, five factors of the [Adolescent Self- rating Life Event Check list](https://www.so.com/link?m=ezLoSRmTisgRPzEzIP6XF5ZaCe4EQ8FUPEnEV6//r8BtRep/lkSKurMA36qi0nlTv0+qS3xE+ymDeV0ouprjrHP4bR2iEpKhxq8wzGUKPHJ/TVjfdkP4cikNKjzsSxBGx29d1hf7ntGx2nys4Mh+GhaN6LejU3grxdTBZ2/FNrqEmesQe5zkeQDHyQvHfs5wBu+/2Xvo/tcUGreHl1mc69/IOLG/YOmS4rrrc4gIlpzsyf1+m6/I3hFMoLI/w2x8P2bWeqk5RIOcqPFPD5KlApQ==).

Table S2. Edge weights matrix for the network of participants without baseline PLEs

|  | PI | BEs | PAs | PHQ1 | PHQ2 | PHQ3 | PHQ4 | PHQ5 | PHQ6 | PHQ7 | PHQ8 | PHQ9 | GAD1 | GAD2 | GAD3 | GAD4 | GAD5 | GAD6 | GAD7 | A1 | A2 | A3 | A4 | A5 |
| --- | --- | --- | --- | --- | --- | --- | --- | --- | --- | --- | --- | --- | --- | --- | --- | --- | --- | --- | --- | --- | --- | --- | --- | --- |
| PI | 0.243 | 0.058 | 0 | 0.028 | 0.032 | 0.026 | 0.041 | 0.025 | 0.031 | 0.026 | 0.016 | 0.009 | 0.017 | 0.017 | 0.025 | 0.022 | 0.008 | 0.016 | 0.015 | 0.002 | 0.091 | 0.111 | 0.133 | 0.111 |
| BEs | 0.062 | 0.222 | 0.007 | 0.013 | 0.020 | 0.015 | 0.013 | 0.009 | 0.012 | 0.026 | 0.012 | 0 | 0.013 | 0.014 | 0.011 | 0.011 | 0.009 | 0.010 | 0.013 | 0 | 0 | 0.011 | 0 | 0.012 |
| PAs | 0 | 0.068 | 0.089 | 0 | 0 | 0 | 0 | 0 | 0 | 0.005 | 0.019 | 0.006 | 0 | 0 | 0 | 0 | 0 | 0.025 | 0.016 | 0.066 | 0.047 | 0.101 | 0.025 | 0.008 |
| PHQ1 | 0 | 0 | 0 | 0.051 | 0.003 | 0 | 0.001 | 0 | 0 | 0.005 | 0 | 0 | 0.005 | 0.005 | 0.003 | 0 | 0 | 0 | -0.028 | 0 | 0 | 0 | 0 | 0 |
| PHQ2 | 0 | 0 | 0 | 0 | 0.045 | 0 | 0 | 0 | 0.011 | 0.014 | 0 | 0 | 0.031 | 0.029 | 0.024 | 0.021 | 0 | 0.029 | 0.022 | 0 | 0 | 0.012 | 0 | 0.123 |
| PHQ3 | 0.048 | 0 | 0.013 | 0.032 | 0 | 0.170 | 0.014 | 0.027 | -0.012 | 0.032 | 0.002 | 0 | 0.011 | 0.024 | 0.010 | 0.042 | 0.025 | 0 | 0.003 | 0 | 0 | 0 | 0.014 | 0 |
| PHQ4 | -0.043 | 0 | -0.018 | 0.051 | 0.041 | 0 | 0.092 | 0 | 0.028 | 0 | 0 | 0 | 0.039 | 0.009 | 0.032 | 0.007 | 0 | 0.006 | 0 | 0 | -0.088 | 0 | 0 | 0 |
| PHQ5 | 0 | 0 | 0.024 | 0.019 | 0.034 | 0.044 | 0.049 | 0.145 | 0 | 0.020 | 0.008 | 0.0002 | 0.011 | 0.038 | 0.014 | 0.037 | 0.012 | 0.033 | 0.031 | 0 | 0 | 0.114 | 0.006 | 0.068 |
| PHQ6 | 0 | 0 | -0.015 | 0 | 0 | 0 | 0 | 0 | 0.107 | 0 | 0 | 0 | 0 | 0 | 0.003 | 0 | 0.027 | 0 | 0.0001 | 0 | 0 | 0 | 0 | 0 |
| PHQ7 | -0.031 | 0 | 0.005 | 0.001 | 0 | 0 | 0 | 0 | 0.014 | 0.132 | 0.002 | 0 | 0 | 0 | 0.008 | 0.001 | 0 | 0 | -0.017 | 0 | 0 | 0 | 0 | 0 |
| PHQ8 | 0.103 | 0.217 | 0.063 | 0.010 | 0.004 | 0 | 0 | 0.020 | 0.033 | 0 | 0.066 | 0.014 | 0.017 | 0.061 | 0.040 | 0.079 | 0.085 | 0.062 | 0.080 | 0 | 0 | 0.067 | 0.062 | 0 |
| PHQ9 | 0 | 0.010 | 0.023 | -0.024 | 0 | 0 | 0 | 0.001 | 0.033 | 0 | 0.022 | 0.172 | 0 | 0 | 0 | 0 | 0.025 | 0.0003 | 0.017 | 0 | 0 | 0.058 | 0 | 0 |
| GAD1 | 0 | 0 | 0 | 0.046 | 0.045 | 0 | 0.013 | 0 | 0.024 | 0 | 0 | 0 | 0.090 | 0.031 | 0.019 | 0.006 | 0.002 | 0.031 | 0 | 0 | 0 | 0.009 | 0.001 | 0.063 |
| GAD2 | 0 | 0.065 | 0 | 0.004 | 0 | 0.009 | 0.005 | 0.009 | 0.013 | 0.018 | 0.015 | 0.006 | 0 | 0.071 | 0.009 | 0 | 0.015 | 0.00000 | 0.021 | 0 | 0 | 0 | 0 | 0 |
| GAD3 | 0.010 | 0.037 | 0 | 0.016 | 0 | 0 | 0 | 0 | 0 | 0 | 0 | 0 | 0 | 0 | 0.029 | 0 | 0 | 0 | 0 | 0 | 0 | 0 | 0 | 0 |
| GAD4 | 0 | 0 | 0 | 0 | 0 | 0 | 0 | 0 | 0.002 | 0 | 0 | 0 | 0.028 | 0.020 | 0.059 | 0.077 | 0 | 0 | 0 | 0 | 0 | 0 | 0 | 0 |
| GAD5 | 0 | 0.042 | 0 | 0.007 | 0 | 0 | 0.013 | 0 | 0.031 | 0.027 | 0.070 | 0.018 | 0 | 0.022 | 0.0001 | 0.015 | 0.103 | 0.029 | 0.043 | 0.078 | 0.008 | 0.167 | 0.121 | 0.035 |
| GAD6 | 0.088 | 0.054 | 0.005 | 0.051 | 0.037 | 0.048 | 0.059 | 0.048 | 0.018 | 0.068 | 0.022 | 0 | 0.032 | 0.031 | 0.014 | 0.058 | 0.034 | 0.128 | 0.031 | 0 | 0.092 | 0.255 | 0.197 | 0.093 |
| GAD7 | 0 | 0 | 0.016 | -0.021 | 0 | 0 | 0 | -0.001 | 0 | 0 | 0 | 0 | 0 | 0 | 0 | 0 | 0 | 0 | 0.058 | 0.136 | 0.168 | 0 | 0.069 | 0.073 |
| A1 | -0.001 | 0 | 0.003 | -0.006 | 0 | 0 | 0 | -0.003 | -0.004 | 0.003 | 0.002 | 0.002 | 0 | 0 | 0 | 0.006 | 0.008 | 0.005 | 0 | 0.190 | 0.008 | 0.003 | 0.037 | 0.033 |
| A2 | 0 | 0 | 0.001 | 0.003 | 0 | 0 | 0 | 0 | 0.005 | 0 | 0 | 0 | 0 | 0 | 0 | 0.001 | 0 | 0 | 0 | 0.027 | 0.232 | 0 | 0.009 | 0.016 |
| A3 | 0.034 | 0.022 | 0 | 0.016 | 0.008 | 0.012 | 0.018 | 0.011 | 0 | 0.005 | 0.003 | 0.004 | 0.011 | 0.006 | 0.006 | 0.00000 | 0.006 | 0.015 | 0.004 | 0.001 | 0 | 0.215 | 0.002 | 0 |
| A4 | 0.011 | 0 | 0 | 0.004 | 0.008 | 0.004 | 0.001 | 0 | 0.014 | 0.005 | 0.008 | 0.002 | 0.003 | 0.002 | 0.007 | 0.001 | 0 | 0.006 | 0.006 | 0.053 | 0.062 | 0.034 | 0.164 | 0.066 |
| A5 | 0.011 | 0.0004 | 0 | 0.005 | 0.007 | 0 | 0.006 | 0.010 | 0.010 | 0.004 | 0 | 0.002 | 0.009 | 0.008 | 0.010 | 0.013 | 0 | 0 | 0.005 | 0 | 0 | 0 | 0.032 | 0.129 |

Note: Variables at wave 2 are in rows and variables at wave 1 are in columns.

PLEs, psychotic-like experiences; PI, persecutory ideation; BEs, bizarre experiences; PAs, perceptual abnormalities; PHQ1-9, 9 items of the Patient Health Questionnaire; GAD1-7, seven items of the Generalized Anxiety Disorder scale; A1-5, five factors of the [Adolescent Self- rating Life Event Check list](https://www.so.com/link?m=ezLoSRmTisgRPzEzIP6XF5ZaCe4EQ8FUPEnEV6//r8BtRep/lkSKurMA36qi0nlTv0+qS3xE+ymDeV0ouprjrHP4bR2iEpKhxq8wzGUKPHJ/TVjfdkP4cikNKjzsSxBGx29d1hf7ntGx2nys4Mh+GhaN6LejU3grxdTBZ2/FNrqEmesQe5zkeQDHyQvHfs5wBu+/2Xvo/tcUGreHl1mc69/IOLG/YOmS4rrrc4gIlpzsyf1+m6/I3hFMoLI/w2x8P2bWeqk5RIOcqPFPD5KlApQ==).
